# Revisiting Risk: Uterine Rupture in a Low-Risk Gravida Without Prior Surgery

**DOI:** 10.7759/cureus.101560

**Published:** 2026-01-14

**Authors:** Vithura Kunarathnam, Mariam Dar, Merrai Asad, Itai Itzhak, Petr Itzhak

**Affiliations:** 1 Obstetrics and Gynecology, Nassau University Medical Center, East Meadow, USA; 2 Medical School, American University of the Caribbean School of Medicine, New York, USA; 3 Obstetrics and Gynecology, St. John's University, New York, USA

**Keywords:** induction of labor (iol), labor augmentation, labor induction, low-risk pregnancy, obstetric emergency, oxytocin, pitocin, spontaneous rupture, unscarred uterus, uterine rupture

## Abstract

Uterine rupture typically occurs in women with prior uterine surgery, but spontaneous rupture in an unscarred uterus is rare. This report highlights such an event in a low-risk gravida following labor induction. This case report is about a 35-year-old gravida 2, para 1 woman at 40 weeks and two days of gestation with no history of uterine surgery underwent labor induction with a Cook balloon catheter and misoprostol. After 15 hours of labor, persistent late fetal heart rate decelerations prompted emergency cesarean delivery. Intraoperative findings revealed a 2-3 cm full-thickness rupture of the anterior uterine wall. Both mother and neonate had favorable outcomes. Spontaneous rupture can occur in unscarred, low-risk uteri. Vigilant fetal monitoring and clinical awareness during labor induction and augmentation are essential for timely diagnosis and intervention.

## Introduction

Uterine rupture is a rare but life-threatening obstetric emergency characterized by a full-thickness disruption of the uterine wall. It most commonly occurs in women with prior uterine surgery, most often from cesarean delivery or myomectomy, and is strongly associated with significant maternal and fetal morbidity and mortality [1,2]. In such cases, the risk is generally recognized and monitored accordingly.

Spontaneous rupture of an unscarred uterus is exceedingly rare, with an estimated incidence of approximately one in 15,000-20,000 deliveries [3,4]. When it does occur, it is often associated with factors such as high parity, uterine overdistension, trauma, obstructed labor, uterine anomalies, or excessive use of uterotonic agents [4]. By contrast, the patient in this case had low parity and lacked these recognized risk factors. In the absence of these risk factors, diagnosis may be delayed, as clinicians may not consider uterine rupture in the differential diagnosis during labor in a low-risk patient.

We report a case of a 35-year-old gravida 2, para 1 woman at 40 weeks and two days of gestation, who presented in early labor with no history of prior uterine surgery, trauma, uterine anomalies, or uterine overdistension. This case highlights the potential for spontaneous uterine rupture to occur unexpectedly in low-risk patients and underscores the importance of maintaining vigilance during routine labor inductions. 

## Case presentation

A 35-year-old gravida 2, para 1 woman at 40 weeks and two days of gestation presented to the Labor and Delivery triage unit on July 1 at 00:30 with complaints of regular uterine contractions that had begun approximately 16 hours earlier. Initially occurring every 10 minutes, the contractions increased in intensity and frequency to every three to five minutes. She denied vaginal bleeding, fluid leakage, or decreased fetal movement.

Her obstetric history included one prior uncomplicated spontaneous vaginal delivery in 2017 in Haiti. Medical history was notable for group B streptococcus (GBS) bacteriuria, sickle cell trait, and gestational glucose intolerance (50 g glucose challenge: 143 mg/dL; three-hour oral glucose tolerance test: 90, 157, 126, 135 mg/dL) (Table 1). She had no prior surgeries and no known family history of obstetric complications.

**Table 1:** Three-hour glucose tolerance test values mg/dL is milligrams per deciliter. Reference ranges based on the Carpenter–Coustan diagnostic criteria.

Time	Values	Reference range
Fasting	90 mg/dL	95 mg/dL
1 hour	157 mg/dL	180 mg/dL
2 hour	126 mg/dL	155 mg/dL
3 hour	135 mg/dL	140 mg/dL

On admission, cervical examination revealed 2 cm dilation, long, and -3 station. Contractions were irregular, and the fetal heart rate tracing was Category I. At approximately 03:45, labor was induced using a Cook balloon catheter and 25 mcg of vaginal misoprostol (Cytotec). At 09:18, she was 4 cm dilated, 80% effaced, and at -2 station, with a category 1 tracing and irregular contractions. The patient requested to ambulate and was allowed to do so.

At 13:50, she was re-examined due to recurrent late decelerations. Cervical exam revealed 5 cm dilation, 80% effacement, and -2 station. Maternal repositioning initially resolved the decelerations to a category 1 tracing. The patient requested epidural analgesia. Subsequent monitoring revealed recurrent late decelerations that were initially responsive to resuscitative measures but ultimately persisted.

After approximately 15 hours of labor, at 17:30, persistent late decelerations unresponsive to maternal repositioning, oxygen, and a fluid bolus prompted emergent cesarean delivery. Intraoperatively, a 2-3 cm full-thickness rupture of the anterior mid-uterine wall was identified, with fetal parts visible through the defect.

A healthy male infant weighing 2905 g was delivered with Apgar scores of 9 at one and five minutes and an umbilical artery pH of 7.28 without evidence of significant acidosis. The mother remained hemodynamically stable throughout the procedure, and the uterine defect was repaired primarily. Postoperative recovery was unremarkable.

## Discussion

Uterine rupture is a critical obstetric emergency typically associated with prior uterine surgery. Spontaneous rupture in an unscarred uterus without identifiable risk factors is rare, with reported incidence ranging from one in 15,000 to one in 20,000 deliveries in high-resource settings. Such cases are often unexpected and can present subtly, challenging clinical detection [1,2].

This case involved a term pregnancy without conventional risk factors. Labor induction followed standard protocols with misoprostol and a Cook balloon catheter, which are considered safe in unscarred uteri. Notably, the patient did not receive oxytocin or undergo amniotomy. Some studies suggest that oxytocin may increase the risk of uterine rupture even in low-risk patients, particularly with higher doses or prolonged use [5]. However, rupture is still most commonly associated with a prior uterine scar. There have been reports of rupture occurring in unscarred uteri with oxytocin administration. In our case, however, the patient did not receive oxytocin, highlighting that spontaneous rupture can occur even in the absence of traditional risk factors and emphasizing the importance of continuous fetal monitoring and clinical vigilance in all laboring patients [6]. 

This case is notable for several reasons that distinguish it from previously reported spontaneous uterine rupture cases in unscarred uteri. Our patient did not receive oxytocin or undergo amniotomy, yet experienced spontaneous rupture, which highlights that such events can occur even in the absence of conventional risk factors or labor augmentation. The presentation was subtle, with recurrent late decelerations as the only abnormal finding without classic symptoms such as abdominal pain, vaginal bleeding, or loss of fetal station, emphasizing the “silent” nature of rupture in low-risk patients. The detailed documentation of the labor course including cervical examinations, fetal heart rate monitoring, and timing of interventions provides a comprehensive picture of the events leading up to spontaneous uterine rupture which is often lacking in prior case reports. Prompt recognition and emergent surgical intervention resulted in favorable maternal and neonatal outcomes. This case challenges existing risk stratification models, suggesting that potential subclinical uterine or myometrial abnormalities that are not detectable with routine clinical evaluation may exist, and reinforces the need for heightened awareness and individualized clinical judgment during labor. Overall, this case demonstrated the need to conduct further research to identify hidden risk factors that could predispose even low-risk patients to uterine rupture. 

Limitations: This single case report limits generalizability. Underlying microscopic uterine anomalies remain speculative, and causality between induction agents and rupture cannot be definitively established. Further research is needed to clarify hidden risk factors and the relationship between labor induction/augmentation and uterine rupture. Larger cohort studies and registry-based analyses, such as those described in the existing literature [3,4], are needed to better characterize induction-related risks in unscarred uteri.

## Conclusions

Spontaneous uterine rupture in an unscarred uterus is rare but can occur even in low-risk pregnancies undergoing labor induction. Continuous fetal monitoring and heightened clinical awareness are essential for early detection and timely intervention. Obstetric providers should balance labor management benefits with vigilance for rare complications to optimize outcomes.
